# Work-Related Mental Health Issues in Graduate Student Population

**DOI:** 10.3389/fnins.2021.593562

**Published:** 2021-04-01

**Authors:** José Ignacio Gallea, Leonardo Adrián Medrano, Luis Pedro Morera

**Affiliations:** ^1^Instituto de Organizaciones Saludables, Universidad Siglo 21, Córdoba, Argentina; ^2^Vicerrectoria de Investigación, Pontifica Universidad Católica Madre y Maestra, Santiago de los Caballeros, Dominican Republic

**Keywords:** graduate students, mental health, well-being, burnout, cortisol, HPA axis (hypothalamus–pituitary–adrenal), engagement

## Abstract

The scientific and educational community is becoming increasingly aware of the impact of current academic working conditions on graduate students’ mental health and how this is affecting scientific progress and ultimately society as a whole. Our study aimed to shed light on the work-related mental health issues affecting graduate students, providing a comprehensive research work including psychological and biological assessment. Our findings showed that a sizeable number of graduate student present anxiety, depression, or high burnout and that the time spent in academia plays an important role. The graduate student population displayed a specific work-related mental health issues profile with an altered hypothalamic–pituitary–adrenal (HPA) axis and low levels of work engagement. Finally, graduate students were equally stressed, with less work engagement, and more anxious and depressed than general workers.

## Introduction

Graduate students in most countries are not considered to be part of the labor force, even though in addition to their degree work they are frequently engaged as research and teaching assistants. These combined responsibilities, along with the psychosocial environment of graduate education, are entailed in the mental health issues associated with work. Burnout, the work-related stress syndrome, is one of these issues (Cornér et al., 2017); it emerges in response to chronic interpersonal stressors on the job and can have a negative influence on an individual’s psychological and physical health (Maslach et al., 2001). Furthermore, the Burnout syndrome may contribute to the development of pathological conditions such as anxiety and depression (Harvey et al., 2013). These two other mental health problems in the workplace are recognized as the leading causes of sickness absence and long-term work disability (Harvey et al., 2013).

Mental health issues in graduate students have been the focus of a few recent investigations, helping to make the problem visible (Levecque et al., 2017; Evans et al., 2018). They show the graduate student population to be at risk of having or developing a common psychiatric disorder (Levecque et al., 2017), with a high prevalence of anxiety and depression (Garcia-Williams et al., 2014; Evans et al., 2018). However, research on the subject remains scarce and, in most cases, does not compare graduate students with the general working population, nor does it include biological assessment. Research on work engagement, a positive work-related state of well-being, has likewise been largely neglected in the graduate student population. This is a critical point since reducing stress or discomfort is not equivalent to increasing well-being (Salanova et al., 2016). Therefore, the assessment of burnout syndrome along with work engagement in the graduate student population emerges as a crucial topic that could be fundamental to the development of early intervention and eventually help to prevent generalized anxiety and depression.

Whereas individual well-being is probably the more important concern, mental health issues could affect the quality and quantity of a researcher’s output, impact the functioning of research teams, and influence entry into and persistence in the research field (Levecque et al., 2017). The combined effect on scientific advancement has high societal costs.

In light of the above, there is an urgent need for more integral studies to better analyze these risk factors with the aim of addressing more effective interventions targeting graduate students’ well-being and health.

In this work, we deployed a range of psychobiological tools to inquire into the work-related mental health issues in a graduate student population. We found that graduate students are deeply affected by these issues and that the accumulation of time spent in academia plays an important role. The graduate student population displayed a specific profile with an altered hypothalamic–pituitary–adrenal (HPA) axis, high levels of burnout, and low levels of work engagement. Finally, graduate students were more affected by these issues than general workers. It is hoped that the data presented here will prompt the international scientific community to take the necessary measures to fully address the issues involved.

## Materials and Methods

### Participants

We conducted a cross-sectional study on a population of science-related graduate students in the field of physics, chemistry, and biology. A convenience sample of 153 Ph.D. students (59% women and 41% men) participated in the study. The sample comprised individuals with an average age of 28 years, SD = 3. All students were enrolled in universities within the authors’ country and recruitment was according to their availability and accessibility. To reduce participation bias, since participants who have had a history of anxiety or depression may be more apt to respond the survey, intense in-person engagement work was done to involve as many students as possible from each research institution visited. For comparison, we surveyed a population of 1,044 Argentinian workers (52% women and 48% men with an age average of 42 years, SD = 13) from diverse fields/types of work (see Supplementary Material) sampled with a probability (cluster) sampling method.

### Procedure

We requested all selected individuals to complete a self-administered questionnaire after reading an accompanying letter explaining the objectives of the study. Informed consent was obtained from all graduate students/participants in the study. In the case of the graduate students, we also provided them with three plastic tubes (Corning LS tubes of 15 ml) and detailed instructions on collecting saliva, emphasizing the need to strictly follow the time schedule and refrain from drinking, eating, and brushing their teeth before collecting the three saliva samples. Inclusion criteria for the study were as follows: representatives of both sexes, appropriate language ability (able to read and complete questionnaires in Spanish), signing of the corresponding informed consent form, and being an active worker with at least 1 months in the job at the time of the sampling. All participants were asked to provide information regarding potential covariates that could affect cortisol levels, considered as exclusion criteria: use of systemic or topical steroids in the last 4 weeks, intense exercise prior to sampling, report of consumption of steroid-based anti-inflammatory drugs, oral injuries or diseases, alcoholism, chemotherapy, prolonged corticotherapy, autoimmune diseases, and infection (Morera et al., 2020). The study had the approval of the Hospital Nacional de Clínicas ethical committee (CIEIS-HNC) of the Faculty of Medical Sciences, National University of Córdoba, which has provincial jurisdiction. Finally, the study was conducted according to the Declaration of Helsinki on studies with human subjects.

### Questionnaires

We provided all participants with a paper-and-pencil questionnaire that included a socio-demographic questionnaire and all the psychological instruments.

The socio-demographic questionnaire included personal details such as gender, age, research field, and starting date of postgraduate education, among other data. We assessed Anxiety and Depression with the brief Patient Health Questionnaire-4 (PHQ-4) (Cano-Vindel et al., 2018). This instrument combines the two short versions (the GAD-2 and the PHQ-2) of the “Generalized Anxiety Disorder–7 scale” (GAD–7) and “Patient Health Questionnaire-9” (PHQ-9) (Kroenke et al., 2009). The Spanish version of the PHQ-4 was validated in our group (Cano-Vindel et al., 2018). GAD-2 and PHQ-2 items were rated on a four-point frequency scale, ranging from one (never) to four (daily). Anxiety and Depression scores for each individual were obtained by adding up the item scores of each respective disorder. We used the reported cutoff of three (Cano-Vindel et al., 2018) in both GAD-2 and PHQ-2 to dichotomize into an Anxiety (A) and non-Anxiety (non-A) symptoms group and a Depressed (D) and non-Depressed (non-D) symptoms group, respectively.

To assess Burnout, we used the Argentinean version (Spontón et al., 2019) of the Maslach Burnout Inventory-General Survey (MB-GS) (Schaufeli and Buunk, 1996; Maslach et al., 2001). This included the exhaustion scale comprising five items (e.g., “I feel emotionally drained by my work”) and the cynicism scale comprising four items (e.g., “I have become less enthusiastic about my work”). We measured work engagement using the Argentinean version (Spontón et al., 2012) of the Utrecht Work Engagement instrument (UWES) (Schaufeli et al., 2002) comprising the vigor scale with six items (e.g., “at my work, I feel bursting with energy”) and the dedication scale, also with six items (e.g., “I am enthusiastic about my work”). MB-GS and UWES items were rated on a seven-point frequency scale, ranging from one (*never*) to seven (*daily*). In order to avoid response bias, we randomly merged all burnout and work engagement items into one questionnaire. Burnout and Work Engagement scores for each individual were obtained by adding up the item scores of each respective instrument. We dichotomized Burnout and work engagement by dividing the MB-GS and UWES total score into Low (≤25th percentile) and high (≥75th percentile). In this way, we obtained the Low Burnout (Low-B) and High Burnout (High-B) group and Low-Engagement (Low-E) and High-Engagement (High-E) group. We used the Low-B and Low-E groups as the reference categories. Dichotomous classification of burnout and work engagement has been used and recommended by different research groups (Ishii et al., 2018; Penz et al., 2018).

### Salivary Cortisol

Salivary cortisol was collected at three time points to determine the cortisol awakening response (CAR): immediately after awakening and 30 and 45 min thereafter (Powell and Schlotz, 2012). All samples were stored at 4°C until sent to the laboratory, where they were then centrifuged for 5 min at 2,000 rpm to extract saliva with low viscosity and subsequently transferred to 1.5-ml tubes and stored in a freezer at –80°C until analysis. After thawing, 20 μl of salivary samples was transferred to a sample cup and cortisol was estimated using a cortisol RP Elecsys kit (Roche Diagnostics, United States) in a cobas e 411 analyzer. Summary indexes of CAR included the area under the curve with respect to ground (AUC_*G*_) and the area under the curve with respect to increase (AUC_*I*_) calculated according to the work of Pruessner et al. (2003).

### Data Analysis

All statistical analyses were performed using SPSS 23.0 (IBM., Chicago, IL, United States). We describe continuous variables using mean with the standard deviation (SD)/standard error of the median (SE) and qualitative variables using frequency (percentage). Skewness and kurtosis were used to test the normality distribution for numeric variables. For univariate analysis, we applied independent *t* test to compare mean values between the two groups. We used the Pearson bivariate correlation to evaluate the linear relationship between month spent in graduate education and the mental health issue scores. In the analytical procedures, a two-sided value of <0.05 was considered as statistically significant. All analyses controlled age, sex, and type of work.

## Results

### Graduate Education: Disadvantages and Drawbacks for Mental Health

With the aim of analyzing the relationship between work-related mental health issues and graduate education, we used a psychobiological approach to assess a cohort of 153 science-related graduate students. Individuals were from the biological, chemical, and physical sciences, 59% women and 41% men, with an average age of 28 years, SD = 3.

**FIGURE 1:**
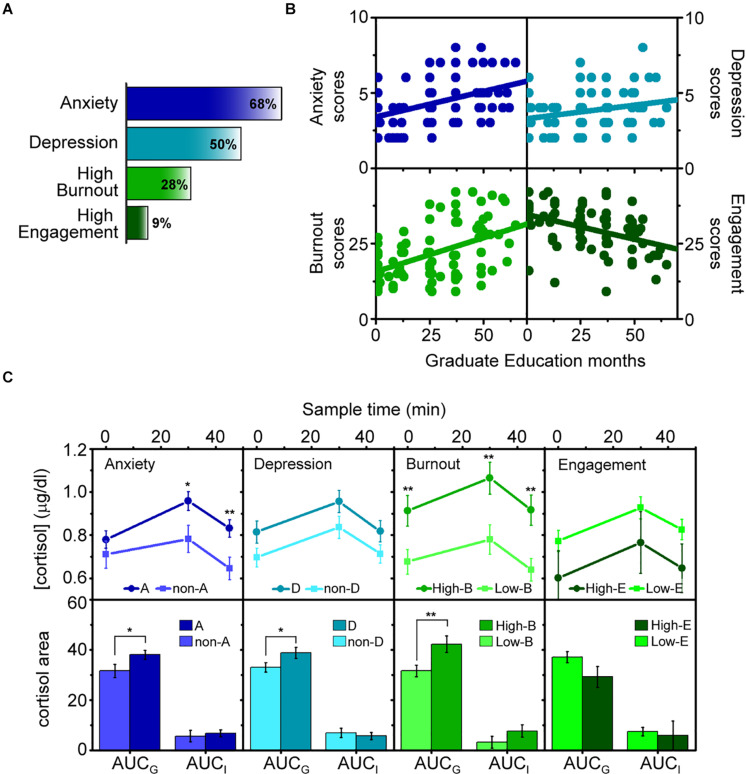
Psychobiological assessment of work-related mental health issues in graduate students. **(A)** Prevalence of the work-related mental health issues within the population of graduate students analyzed. **(B)** Linear correlation of months of graduate education with Anxiety (Pearson *r* = 0.337, *p* = < 0.001), Depression (Pearson *r* = 0.195, *p* = < 0.05), Burnout (Pearson *r* = 0.454, *p* = < 0.001), and Work Engagement (Pearson *r* = −0.424, *p* = < 0.001) scores among the students. **(C)** Comparison of the cortisol awaking response (CAR) and its respective indices (AUC_*G*_ and AUC_*I*_) between the different graduate student groups, i.e., graduate students with (A) and without anxiety symptoms (non-A), with (D) and without (non-D) depression symptoms, with High (High-B) and Low (Low-B) Burnout, and with High (High-E) and low (Low-E) work engagement. The graphs show mean values, with error bars representing the standard error of the mean. Statistical significance: *p < 0.05; **p < 0.01.

We relied on psychological tools to address a comprehensive study of the work-related mental health issues prevalence in this population and their relationship with graduate education. We included an anxiety and depression assessment and also Burnout and Work engagement, two of the lesser studied issues in this population. Based on the cutoff value of three for the GAD-2 anxiety scale and PHQ-2 for the depression scale (Cano-Vindel et al., 2018), we found a prevalence of 68% for anxiety symptoms and 50% for depression symptoms (Figure 1A). In line with the results of Evans and co-workers, our findings show that these common mental issues have a high prevalence in graduate students. Differences in the percentages between the two studies may be related to the sample characteristics or the specificity/sensitivity relation of the psychological instrument. As shown in Figure 1B, the Pearson correlation statistic showed a significant positive correlation between the time elapsed in graduate education and both anxiety and depression scores. These data suggest that graduate education is positively related to these issues.

Various studies have shown that graduate students commonly feel stress and exhaustion, among other negative symptoms (Hyun et al., 2007; Cornér et al., 2017). It was reported that a population of graduate dental students presented high rates of burnout symptoms, particularly emotional exhaustion and reduced personal accomplishment (Divaris et al., 2012). We therefore analyzed the prevalence of burnout in this graduate student population. We measured the manifestation of emotional exhaustion and cynicism toward their work, two of the main domains of burnout present in the Maslach Burnout Inventory (MBI) (Spontón et al., 2019). The scores obtained were added up and expressed as the burnout score. As in studies by other groups, further dichotomization was used to categorize into low burnout vs. high burnout (see section “Materials and Methods”). We found that an appreciable amount of students (28%) (Figure 1A) presented high burnout and that burnout scores tended to be higher as the months of graduate education increased (Figure 1B). We analyzed work engagement in the same manner. Work engagement is understood as a positive work-related affective–cognitive state of mind characterized by the manifestation of vigor and dedication in one’s work (Attridge, 2009). It is important to measure work engagement independently of burnout since it is not considered to be an exact counterpart to the latter (Schaufeli and Salanova, 2011). We obtained the work engagement score as the sum of the measured vigor and dedication scores from the Utrecht Work Engagement Scale (UWES) (Spontón et al., 2012), categorizing them into low and high work engagement. As shown in Figures 1A,B, only 9% of graduate students presented high work engagement and there is an inverse correlation between the months spent in graduate education and the work engagement score. Collectively, the data presented above demonstrate that work-related mental health issues favored by graduate student education have a considerable prevalence in the graduate student community, to the detriment of well-being.

### Glucocorticoid Dysregulation in Graduate Students

Deregulation of the HPA axis by the work-related mental health issues analyzed in this work has been widely demonstrated. For instance, symptoms for anxiety and depressive disorders have been linked with hypersecretion of corticotropin-releasing hormone (CRH) and high levels of circulating glucocorticoids (Gold et al., 2002; Schulkin, 2011). However, high levels of cortisol have been positively associated with burnout syndrome (Penz et al., 2018), this finding being in dissonance with other studies reporting a negative relationship between these variables (Oosterholt et al., 2015; Pilger et al., 2018). In addition, differences in cortisol suppression in response to the dexamethasone suppression test were found between burnout and engaged workers, indicating a higher HPA axis feedback sensitivity for the latter (Langelaan et al., 2006; Bakker et al., 2011). Nevertheless, only few biological assessments have been conducted on the graduate student population. Ignacchiti and co-workers showed high plasma cortisol concentration in graduate students with pre-final examination anxiety (Ignacchiti et al., 2011), while flattening of cortisol awaking response (CAR) was determined before the graduate education entrance exam (Duan et al., 2013) and after the first year (McGregor et al., 2016). To further analyze how work-related mental issues in graduate students are associated with dysregulation of their HPA axis, we measured the CAR in this population. A plethora of studies have suggested the CAR as a standard tool for testing HPA axis integrity (Fries et al., 2009). To determine CAR, salivary cortisol was sampled at three time points, immediately upon awakening (CORT-1) and 30 (CORT-2) and 45 (CORT-3) min later (Powell and Schlotz, 2012). Cortisol was compared between groups of graduate students with different disorders and their counterparts (Figure 1C). All three cortisol measures as well as the widely used and recommended summary indexes of CAR, i.e., the AUC_*G*_ and the AUC_*I*_ (Pruessner et al., 2003), were contrasted. While AUC_*G*_ is related to total hormonal output, AUC_*I*_ emphasizes changes over time and is associated with the sensitivity of the system (Fekedulegn et al., 2007). Compared to the non-Anxiety group (non-A), results show significantly higher mean values for COR-2, CORT-3, and AUC_*G*_ in the Anxiety group (A) (Figure 1C), indicating an altered cortisol secretion immediately after awaking. A cortisol increase was also found in the depression (D) and the high burnout (High-B) group with a significant value in AUC_*G*_ for the former and a more marked significant difference in COR-1, CORT-2, CORT-3, and AUC_*G*_ for the latter (Figure 1C). In the High-B group, the higher level of CORT-1 indicates a dysregulation of cortisol that may reflect altered nocturnal secretion patterns in individuals with these issues. In this regard, it has been shown that CORT-1 levels reflect secretory activity during the late stages of sleep (Wilhelm et al., 2007). High levels of cortisol in the inactive/night period were associated with stress-related sleep deprivation, acting as a risk factor for insulin resistance (McEwen, 2006). It is worth mentioning that AUC_*I*_ mean values were not significantly different between these groups, demonstrating that changes in the total amounts of the hormone were not accompanied by changes in cortisol profiles and in rates of change (Fekedulegn et al., 2007).

Finally, we found an opposite trend in work engagement, but the differences with their counterparts were not significant. This is in agreement with the idea that engaged employees need to mobilize resources to face job demands, giving rise to a state of activation–tension where cortisol may participate in an orchestrated manner.

### Graduate Students Are More Affected by These Issues Than the General Working Population

**FIGURE 2:**
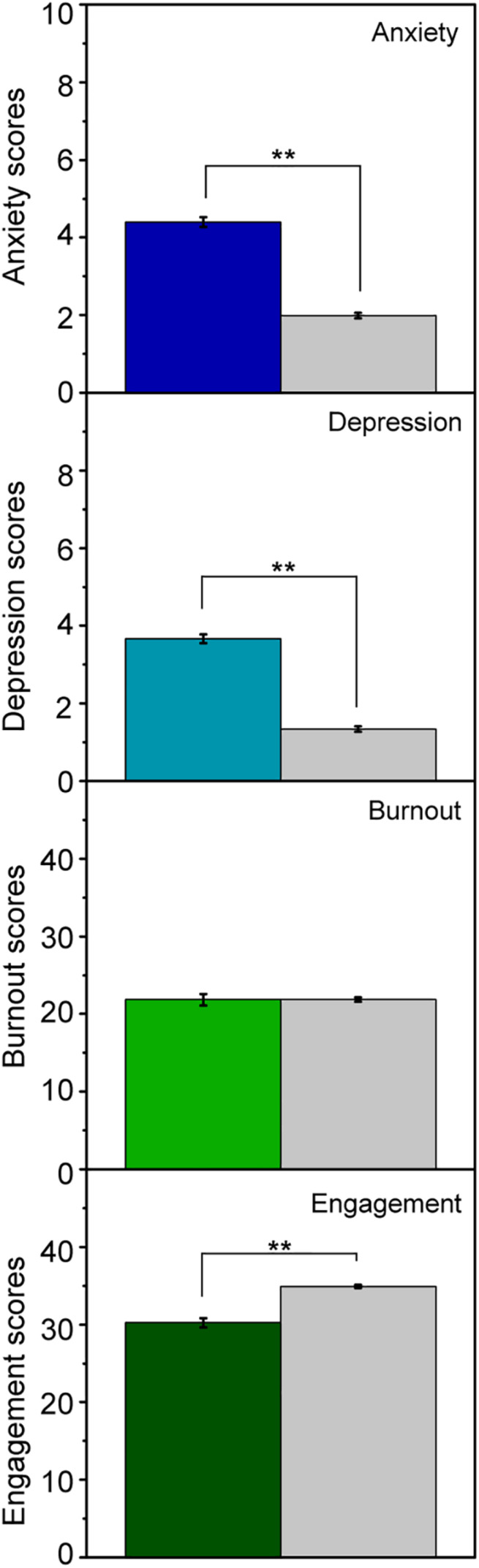
Graduate student vs. general worker population. Comparison of the work-related mental health issues scores between the graduate student population (color bars) and the general working population (gray bars). The graphs show mean values, with error bars representing the standard error of the mean. Statistical significance: **p < 0.01.

To determine whether the psychosocial conditions of graduate education are as detrimental as those of works in general, we carried out a first-time comparison between graduate students and a population of 1,044 employees. The employees analyzed were from diverse fields/types of work, ages, and genders. The results of the comparative analysis between the two groups in terms of the mental health issues cited above are shown in Figure 2. The scores were significantly higher in the graduate student group for Anxiety and Depression symptoms, indicating that, in line with previous research (Evans et al., 2018), this population is more likely to experience these two common mental health issues. The differences observed between the two population were independent of age, sex, and type of work. A comparison of burnout scores showed no significant differences between the two populations (Figure 2), indicating that graduate students experience similar work-related stress to general workers.

Work engagement encompasses dedication, enthusiasm, inspiration, and pride, in addition to a sense of meaning and being challenged by one’s work. Individuals with low engagement have a worrying psychological profile, with lower levels of energy and mental resistance and a lower desire to invest effort in their work. Despite the importance of work engagement, to the best of our knowledge, no previous study has measured it in the graduate student population. Surprisingly, our evaluation of work engagement in this population shows a significantly lower mean score than in the case of the working population in general (Figure 2).

Overall, our findings show that the graduate education environment is more detrimental to mental health than general working conditions and is also unfavorable to well-being.

## Discussion

In recent years, there has been a growing awareness that not all occupational hazards have a physical occurrence. Substantial scientific evidence indicates that psychosocial factors can deeply affect the physical and mental health of workers. In this regard, the World Health Organization has underlined the need to strengthen efforts to extrapolate the impact of psychosocial risk on different health outcomes with the aim of improving the health of workers around the globe (Leka et al., 2010). This is particularly important in the context of the scientific community and in particular graduate students, who are not immune to these problems; research is required with a specific focus on their particular characteristics and environment. Our study aimed to shed light on the work-related mental health issues affecting graduate students, providing a comprehensive study including psychological and biological assessment. We show that a sizeable number of graduate student present anxiety, depression, or high burnout and that these disorders are accompanied by the unbalanced secretion of cortisol. This is critical due to the implication of cortisol, as a biological intermediary, in different medical conditions including metabolic syndrome, cancer, obesity, cardiovascular disease, and increased susceptibility to infections (Russell and Lightman, 2019). The association we found between months spent in graduate education and these work-related mental health issues indicates the decisive role that graduate education plays in these disorders and opens up a novel scenario for research focus. Furthermore, we found that graduate students are equally stressed and more anxious and depressed than general workers.

Bearing in mind that well-being is not equivalent to reduced discomfort, we evaluated work engagement in graduate students. Our findings show a negative impact of graduate education on work engagement, the latter being lower in the graduate student population than among general workers. Empirical research shows that workers with vigor and dedication, two key aspects of work engagement, are able to create their own resources, helping them to perform better (Bakker et al., 2008). Promoting work engagement may not only benefit graduate students’ well-being but also enhance their scientific output.

Taken together, these findings highlight the importance of addressing work-related mental health issues in graduate students, a deeply affected segment of the population, and serve toward the implementation of decision-making processes and new policies. In line with this, it is also necessary to empirically determine the psychosocial factors that influence well-being in graduate education. Some of these factors are perhaps what we like to call “diseases of science,” such as the publish or perish culture (Kiai, 2019; Poppelaars et al., 2019) or the replication crisis (Pashler and Wagenmakers, 2012; Smith, 2017), that affect the scientific community in general and the progress of science. In this regard, a recent survey of more than 4,000 scientists indicates that 55% have a negative impression of research culture, and one-quarter said that the culture damaged the quality of research (Abbott, 2020). It is most likely that additional factors specific to the graduate student population are also at play, such as mentorship relationships (Evans et al., 2018), job demands (Levecque et al., 2017), etc. Are all these psychosocial factors promoting work-related mental health issues? Is there a vicious cycle of factors and disorders? Can all this generate a distinct pattern of comorbidity leading to a more complex “Science disease”? These are all questions that need to be answered.

## Data Availability Statement

The raw data supporting the conclusions of this article will be made available by the authors, upon reasonable request.

## Ethics Statement

The studies involving human participants were reviewed and approved by Hospital Nacional de Clínicas ethical committee (CIEIS-HNC) of the Faculty of Medical Sciences, National University of Córdoba. The patients/participants provided their written informed consent to participate in this study.

## Author Contributions

LM conceived the project. JG and LM conducted the experiments. JG analyzed the data and wrote the manuscript. All authors discussed the results and read and approved the final manuscript.

## Conflict of Interest

The authors declare that the research was conducted in the absence of any commercial or financial relationships that could be construed as a potential conflict of interest.
