# Supplementary material for: Work-Related Mental Health Issues in Graduate Student Population
Source: Front Neurosci. 2021 Apr 1;15:593562. doi: 10.3389/fnins.2021.593562 (PMC8049290; doi:10.3389/fnins.2021.593562)
Supplement: Supplementary file 1 [file Table_1.DOCX]

Supplementary Material

**Supplementary Table 1.** Frequency distribution of workers by demographic profile.

|  | **Frequency (*n*)** |
| --- | --- |
| **Ocupational Sector** |  |
| Public | 9 % (94) |
| Private | 54.4 % (568) |
| Self-employed professional | 15 % (157) |
| Self-employed | 12.9 % (135) |
| Other | 8.6 % (90) |
| **Sector** |  |
| Industry | 11.9 % (124) |
| Commerce | 20 % (209) |
| Service | 66.8 % (697) |
| Agriculture | 0.9 % (9) |
| Do not know/do not answer | 0.5 % (5) |
| **Type of company** |  |
| Micro (<10 employees) | 22.5 % (235) |
| Small (10-50 empleyees) | 41.4 % (432) |
| Medium (50-250 employees) | 13.4 % (140) |
| Large (>250 employees) | 20.4 % (213) |
| Do not know/do not answer | 2.3 % (24) |
|  |  |
